# Specific Genotypes Associated with Differences in Fasting Insulin Levels and Body Mass Index in Healthy Young Males: Implications for Gene–Nutrient Interactions—an Exploratory Study

**DOI:** 10.1016/j.cdnut.2023.102018

**Published:** 2023-10-21

**Authors:** Julie E. Brown, Toan Pham, Hannah Burden, Andrea J. Braakhuis

**Affiliations:** 1 The Discipline of Nutrition, School of Medical Sciences, Faculty of Medical and Health Sciences, The University of Auckland, Auckland, New Zealand; 2 Auckland Bioengineering Institute, The University of Auckland, Auckland, New Zealand

**Keywords:** genetic variation, gene–nutrient interaction, interindividual differences, nutrigenomics, single nucleotide polymorphisms

## Abstract

**Background:**

Genetic variation may significantly impact an individual's susceptibility to diseases, particularly when combined with specific nutrients. Additionally, genetic variations can lead to interindividual differences in metabolic responses.

**Objective:**

The present study explores the association between gene variants and observed interindividual differences in metabolic responses.

**Methods:**

The study included 30 healthy males (aged 20–34) who underwent a fasting period and subsequently consumed a standardized meal. Blood samples were collected both before and after the meal to assess metabolic changes. BMI served as an indirect measure for assessing physiological responses associated with body composition. Appetite changes were assessed using an online Visual Analog 100-point Scale. Buccal swabs were collected to analyze genetic variants in single nucleotide polymorphisms (SNPs).

**Results:**

The data underwent multiple regression analysis, revealing significant associations with 3 SNPs and their metabolic status: the insulin-receptor substrate 1 (*IRS1*) gene variant rs2943641, genotypes CT and CC, with elevated fasting insulin levels (*R*^*2*^ = 0.639, *P* = < 0.0001); the mitochondrial uncoupling protein 1 (*UCP1*) gene variant rs1800592, genotypes GG and GA, with increased BMI (*R*^2^ = 0.261, *P* = 0.007); and the peroxisome proliferator-activated receptor γ2 (*PPARγ2*) gene variant rs1801282, genotypes GG and GC, with increased BMI (*R*^*2*^ = 0.200, *P* = 0.024).

**Conclusions:**

Therefore, our study established significant associations between these 3 SNPs and differences in fasting insulin levels and BMI within our cohort.

## Graphical abstract

**Figure:**
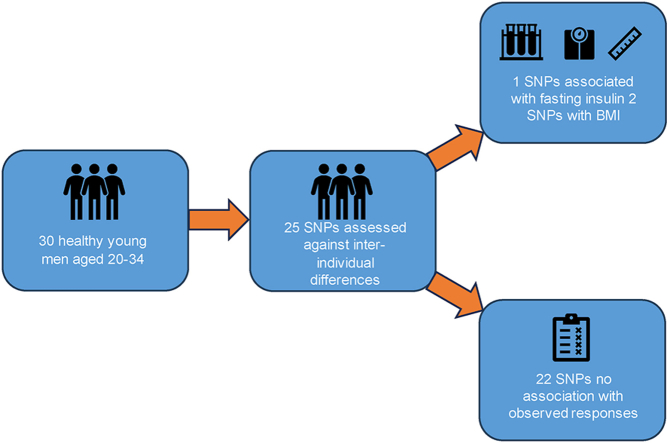

## Introduction

A genetic and nutrient-based approach has been proposed for tailoring dietary requirements to maintain optimal health [1, 2]. However, despite the growing acknowledgment of gene–nutrient interactions, there is still limited understanding in this field. For instance, when it comes to individual dietary advice for the management of diseases associated with hyperglycemia or hyperlipidemia, the advice is often uniform regardless of diverse health histories [3, 4]. Additionally, research suggests that certain individuals may not experience the expected health benefits from these general dietary recommendations [5, 6]. These findings challenge population-level guidance as there are considerable interindividual differences in how individuals respond to specific diets [[7], [8], [9], [10], [11], [12], [13], [14], [15], [16]].

Emerging research consistently reports the importance of nutrition therapy in effectively reducing risk of many chronic diseases, such as type 2 diabetes [11], cardiovascular disease [12], liver cirrhosis [17], obesity [7], and enhanced all-cause mortality in both type 2 diabetes [9] and cancer [18]. However, to optimize nutrition therapy, precision nutrition is required, as it integrates genetic testing to account for the interindividual differences that exist when implementing interventions [[19], [20], [21]]. Therefore, well-designed gene–nutrient studies are essential in providing robust evidence that helps tailor diets to accommodate interindividual differences, thereby promoting optimal health benefits [22, 23].

Single nucleotide polymorphisms (SNPs) are one aspect of genetic variability that can impact interindividual differences among individuals. For example, how individuals respond to different diets can be influenced by their metabolic efficiency and subtle genetic variations associated with lipid metabolism, glucose metabolism, insulin response, and appetite regulation [[24], [25], [26], [27]]. These variations result from distinct allelic variants derived from genetic polymorphisms, with the most prevalent form being site-specific variations known as SNPs [28]. Consequently, SNPs can serve as nutrient-related genetic markers to enable the study of associations between genetic variations and metabolic responses influenced by dietary compositions [29, 30].

The human genome exhibits over 10 million SNPs that vary between individuals [31]. Genome-wide association studies (GWAS) have identified interactions between SNPs and nutrients, revealing increased susceptibility to disease in individuals with certain genotypes [2, [31], [32], [33]]. For instance, the association of the *GLUT2* gene variant rs5400 with glucose homeostasis and insulin release during the postprandial state. This gene variant increases risk of type 2 diabetes, especially among individuals with genotype TT or TC who have a greater desire for sugary foods [[34], [35], [36]]. Other research has focused on genetic susceptibility and eating behaviors contributing to obesity [37]. However, the robustness of gene–nutrient interactions still requires further investigation [20, 21]. Translating GWAS findings into clinically meaningful dietary advice poses challenges, necessitating follow-up studies to determine the magnitude of these associations.

This study presented an opportunity to conduct significant research to enhance our understanding of gene–nutrient interactions by utilizing existing data from the main extensive study documented in the study design. This study's objectives centered around conducting a comprehensive analysis of genetic markers linked to nutrient metabolism and their potential association with key metabolic responses. These responses encompassed fasting, postprandial fatty acid responses, appetite, and variations in BMI. Our overarching goal was to investigate the impact of genetic variations and ascertain whether they were significantly associated with differences in metabolic responses among the participants, depending on their genotypes.

The decision to use a cohort of 30 participants was based on the extensive data collected from the same number of eligible participants in the main investigation and the standardized list of SNPs measured by Nutrigenomix, a biotechnology company. Nutrigenomix produced personalized nutritional reports that detailed nutrient metabolism based on a specific set of SNPs. This allowed us to focus our research on the specific SNPs in this report.

In summary, this study aimed to investigate the significant association between genetic variability and both physiological and subjective responses before and after consuming a standardized meal in a cohort of 30 participants. The analysis focused on 25 specific nutrient-related SNPs. Physiological responses included fasting responses (vitamin D, iron, zinc, low-density lipoprotein (LDL), high-density lipoprotein (HDL), total cholesterol, triglycerides (TG), glucose, and insulin concentrations) and postprandial fatty acid responses were assessed through blood assays. BMI was also measured. Subjective responses regarding appetite changes were assessed using a questionnaire. Our hypothesis proposes that genetic variations will be associated with differences in metabolic responses among the participants.

Although using fasting data would have sufficed, considering postprandial fatty acid responses may be linked to metabolic disorders such as dyslipidemia, and alterations in appetite can indirectly reveal variations in metabolic regulation, affecting factors such as nutrient intake and energy balance [22, 24, 37, 38]. BMI served as an indicator of body composition, which can be influenced by metabolic processes [23, 26]. Although not being traditional or direct metabolic parameters, postprandial fatty acid responses, appetite changes, and BMI can serve as valuable indicators or markers of metabolic function, especially when examined in the context of meal responses [22, 37, 38].

## Methods

### Study design

**FIGURE 1:**
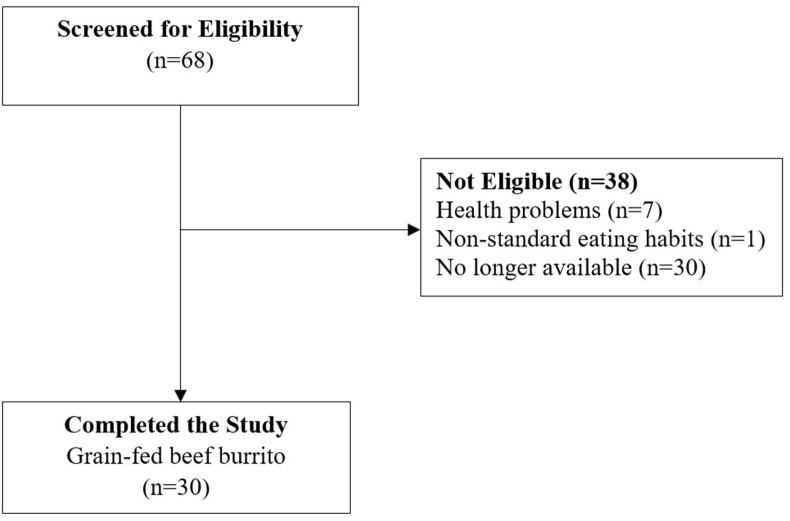
Flowchart of the study participants recruitment (30 healthy young males).

Thirty healthy young males (20–34 y) representing diverse ethnicities participated in a standardized fasting period and subsequently consumed a standardized meal (Figure 1, Supplemental Table A.1). Blood samples and questionnaires were collected before and hourly for 4 h after the meal. Research suggests that most lipid changes occur within 4 h of a meal, capturing the peak lipid responses, with TG and cholesterol reaching their peaks within 2 to 4 h [38].

The study was conducted between October and December 2020 at the University of Auckland Clinical Research Center. The clinical trial, Ref: NCT04545398, was approved by the New Zealand Ministry of Health’s Health and Disability Ethics Committees (Ref: 19/STH/226) and conducted following the ethical standards of the 1964 Declaration of Helsinki. Informed consent of each participant in the study was obtained.

This study constitutes a small subsection of a more extensive investigation [39]. The overarching investigation employed an acute, blinded, randomized cross-over design intervention aimed to compare the effects of beef, lamb, and a meat analog on digestive, metabolic, and nutritional outcomes, 31 May 2022, PREPRINT (Version 1) available at Research Square (doi.10.21203/rs.3.rs-1640468/v1). The extensive investigation was registered under the universal trial number U1111-1244-9426, the first registration was on 11/09/2020. The clinical trial prerecruitment (Ref: NCT04545398) was conducted under the auspices of the University of Auckland. In this specific study, we utilized the sample size of 30 participants (Figure 1) used from the extensive investigation [39] and utilized the data collected from the 4 different mixed meals. Given the substantial similarity of the data across these different meals, we randomly selected and analyzed the data from the grain-fed beef mixed meal for the purpose of this study.

### Eligibility criteria

All participants were omnivores willing to consume a standardized meal. Eligible participants completed an online screening, which included a health survey. Males were recruited from the millennial generation (20–34 y), as males typically have a greater postprandial lipid response than females [29]. The study excluded participants with chronic health conditions, hyperlipidemia (elevated concentrations of plasma cholesterol and TG), BMI ≥ 30 kg/m^2^, use of medications (except occasional use of nonsteroidal anti-inflammatory drugs and antihistamines), history of anosmia and ageusia (issues with taste and smell), current dieting or disordered eating pattern, smokers, and recreational drug users.

### Meal preparation

The standardized meal consisted of 2 hot burrito wraps with meat, vegetables, and sauce, served in the morning after an overnight fast. The meat was a 220 g raw serving of minced beef (approximately 160 g cooked). The standardized meal was prepared according to a uniform recipe specifically created by our research dietitians (meal contained 2940 kJ, 61 g protein, 30 g fat, and 71 g carbohydrate, Table 1). The meal was then cooked fresh and served at the test kitchen site at the University of Auckland Clinical Research Center. The recipe was analyzed using the New Zealand food database for macronutrient contents using Foodworks 10 Professional software (Xyris). The meat was grain-fed New Zealand Angus steer beef, specifically slaughtered, minced to ensure homogeneity and for more rapid digestion [40], packaged, and stored at the research center. All other food items were purchased at a local supermarket.

**TABLE 1:** Nutritional value of the standardized meal based on the standardized recipe

Nutrient	Nutrient value
Energy (kJ)	2940
Protein (g)	61
Fat (g)	30
Saturated Fat (g)	12
Carbohydrates (g)	71
Fiber (g)	29
Sodium (mg)	983

Each participant had the same standardized meal prepared in the university kitchen. Salter scales were used to measure the exact quantities per person: 220 g of mincemeat, 54 g of chopped brown onion, 72 g chopped red bell pepper, 67 g of canned corn kernels, 2 jumbo tortilla wraps, 1/6 jar of salsa sauce, and salt and pepper for seasoning. The onions were fried in a teaspoon of oil, using an electric wok, until tender, before adding the mincemeat. Cooking of the mincemeat continued until it reached a temperature of at least 70°C (checked with a PUREQ solo probe food thermometer). Next was the addition of the bell pepper, corn, salsa, and seasoning. The meal was left to simmer for 10 min before equally dividing the mixture into 2 portions onto 2 flat tortillas. The tortillas were folded and placed under a sandwich grill until toasted and then wrapped in aluminum foil and served. The nutritional value of the standardized meal is provided in Table 1, and the nutrient composition per 100 g of the cooked meal has been previously reported [39].

### Data collection

Participants were asked to maintain a normal lifestyle and physical activity schedule before the study. Reminder text messages were sent to participants the night before the visit to ask them to fast from 22:00 (only water was allowed for the rest of the evening). The participants arrived at the clinical research facilities at 07:30, and their baseline data was collected before meal consumption. A wall-mounted stadimeter was used to measure the participant’s height with their shoes off, and a digital scale was used to measure body weight. Participants were asked to rest for 15 min before their blood pressure and heart rate measurements were taken using an automatic blood pressure monitor (Omron HEM-7130). Buccal swabs were collected and sent for analysis.

A cannula was inserted into the antecubital vein of the forearm. Venous blood was collected into ethylenediaminetetraacetic acid (EDTA)–containing tubes immediately prior to the meal (premeal) and hourly for 4 postprandial hours. Participants were instructed to consume the provided meal within 15 min. Blood samples were centrifuged at 1,500 g for 15 min at 4°C. An aliquot of plasma was maintained at 4°C for the chylomicron-rich fraction (CMRF) isolation protocol. The remaining plasma samples were stored at -80°C for later analysis.

Appetite changes were assessed using an online Visual Analog 100-point Scale (Qualtrics software, SAP). The questions covered fat-taste perception, sugar preference, and hunger, which had previously been validated for use in single-meal investigations [41]. The questions asked included: would you like something fatty to eat? (Strongly agree—0 to strongly disagree—100); would you like something sweet to eat? (Strongly agree—0 to strongly disagree—100) and how hungry do you feel? (Extremely likely—0 to extremely unlikely—100).

The participants completed the questionnaire upon arrival, immediately after, 30 min after meal consumption, and hourly for 4 h. A postmeal survey was sent to all participants at the end of the test day to enquire whether participants experienced any adverse side effects.

### Genetic analysis

Isohelix SK 5mL buccal swab kit and Dri-Capsule were used to collect genetic samples. The participants, in a fasted state, rubbed both of their inside cheeks for 20 s on each side. The buccal swab was then placed immediately into a 5ml tube, and a Dri-Capsule was added. Each collection tube was labeled participant’s research number and a unique identification number. The samples were sent to the Nutrigenomix laboratory, a CLIA-certified and CAP-accredited (College of American Pathologists) facility at the University of Sydney, Australia. Genotype was conducted using the iPLEX Gold assay with mass spectrometry-based detection on the Sequenom MassARRAY platform (Agena Bioscience) [42]. The Illumina assay technique was used to identify DNA mutations, including different insertions of the addition or deletion of a base for risk variant determination [43].

### Digestive and biochemical analysis

The CMRF were extracted from plasma samples for the analysis of intake of long-chain PUFA (LCPUFA), as previously described [44]. Briefly, 3.5 mL of plasma was overlaid with 1.2 mL of saline solution (d =1.006 g/mL) in an OptiSeal Polypropylene tube (Beckman Coulter Life Sciences). After its cap was carefully sealed, the tubes were placed in a TLA-110 rotor (Beckman Coulter) and centrifuged at 117,000 g for 10 min in an Optima MAX-XP ultracentrifuge (Beckman Coulter). The visible top layer was aspirated into new microcentrifuge tubes and corrected to a final volume of 1.4 mL using saline solution. This step provided a standardized dilution factor of the collected CMRF volume relative to the initial plasma volume, and collected CMRF samples were stored at -80°C until further analysis. The CMRF samples were sent to AgResearch Ltd to analyze the fatty acid composition using the fatty acid methyl esters assay previously described [44].

Plasma concentration of vitamin D was analyzed using ultra-high-performance liquid chromatography-tandem mass spectrometry (UHPLC-MS/MS) as previously described [45].

The inductively coupled plasma mass spectrometry (ICP-MS) was used to analyze iron and zinc plasma concentrations at Analytica Laboratories, Hamilton, New Zealand. The plasma samples were digested in aqua regia on a hot block for 2 h. After digestion, Type 1 water was added to dilute the sample 50-fold. Samples were analyzed on a Perkin Elmer ICP-MS fitted with a CETAC autosampler. The internal standard and carrier solutions were introduced into the instrument using an ESI peristaltic pump and were combined with the sample before injection into the instrument by the nebulizer. The plasma was formed using argon gas. Both standard and kinetic energy discrimination (KED) modes were used, with helium gas being introduced into the collision cell for the operation of KED mode to remove polyatomic interferences where required. The instrument was calibrated using a 1-point calibration, and this calibration was verified with a range of internal quality controls.

Plasma concentrations of insulin, glucose, total cholesterol, HDL, LDL, and TG were analyzed using Roche Cobas C311 and E411 autoanalyzer and commercially available Cobas Elecsys assays (Roche Diagnostics).

### Data associations and justification

The selected SNPs have been previously identified and reported to be associated with physiological or subjective responses aligned with our tested protocols [46]. The justification for the association between these SNPs and the related physiological and subjective responses is based on gene–nutrient interaction studies [46], presented in Table 2.

**TABLE 2:** Investigated physiological and subjective measures with the associated SNP justified alongside the published gene–nutrient interaction

Associated gene	Published gene–nutrient interaction^a^	Gene variant	SNP reference	Physiological & subjective measures
Cytochrome P450 family 2 subfamily R member 1	Involved in activation of vitamin D	CYP2R1	rs10741657	Vitamin D ng/mL premeal
Group-specific component vitamin D binding protein	Involved in vitamin D transport	GC	rs2282679	Vitamin D ng/mL premeal
Homeostatic iron regulator protein	Involved in iron transport	HFE	C282Y rs1800562 H63D rs1799945	Iron μmol/L premeal
Solute carrier family 17 member	Involved in iron transport	SLC17A1	rs17342717	Iron μmol/L premeal
Transmembrane protease serine 6	Involved in absorption of iron	TMPRSS6	rs4820268	Iron μmol/L premeal
Type-2 transferrin receptor	Involved in iron transport	TRF2	rs7385804	Iron μmol/L premeal
Transferrin coding	Involved in iron transport	TF	rs3811647	Iron μmol/L premeal
Solute carrier family 30 member 3	Involved in zinc transport	SLC30A3	rs1126936	Zinc mg/L premeal
Fatty acid desaturase 1	Involved in synthesis of LCPUFA 18:2 n-6 &18:3 n-3	FADS1	rs174546 & rs174547	LCPUFA^b^mmol/L changes (AUC^c^)
Mitochondrial uncoupling protein1	Linked to body weight	UCP1	rs1800592	BMI kg/m^2^
Transcription factor 7-like2	Linked to body weight	TCF7L2	rs7903146	BMI kg/m^2^
Apolipoprotein A-II	Involved in response to intake of saturated fat	APOA2	rs5082	LDL mmol/L premeal
Fat-mass and obesity-related alpha-ketoglutarate-dependent dioxygenase	Involved in response to intake of fats and body weight	FTO	rs9939609	BMI kg/m^2^
Fat-mass and obesity-related alpha-ketoglutarate-dependent dioxygenase	Linked to body weight	FTO	rs9939609	BMI kg/m^2^
Peroxisome proliferator-activated receptor γ2	Linked to body weight	PPARγ2	rs1801282	BMI kg/m^2^
Apolipoprotein A5	Involved in lipid metabolism	APOA5	rs662799	Total cholesterol premeal
ATP-binding cassette subfamily G member8	Involved in cholesterol uptake	ABCG8	rs6544713	LDL mmol/L premeal
ATP-binding cassette subfamily A member 1	Involved in cholesterol metabolism	ABCA1	rs1883025	HDL mmol/L premeal
Angiopoietin-like 3	Involved in release of fatty acids and glycerol from adipose tissue	ANGPTL3	rs10889353	Triglycerides mmol/L premeal
Cluster determinant 36	Involved in lipid absorption and response to fat detection	CD36	rs1761667	Fat-taste(AUC) perception changes
Glucose transporter type 2	Involved in glucose transport and insulin release	GLUT2	rs5400	Sugar preference changes (AUC)
Neuromedin beta	Involved in eating behaviors	NMB	rs1051168	Hunger changes (AUC)
Adenylate cyclase 5	Involved in glucose levels	ADCY5	rs11708067	Glucose mmol/L premeal
Insulin-receptor substrate 1	Involved in insulin-signaling pathway	IRS1	rs2943641	Insulin μU/mL premeal

a Published gene–nutrient interactions listed in the table have been suggested by previous genome-wide association studies to be associated with disease risk in a population [46]

b LCPUFA, long-chain PUFA, linoleic acid (LA) C18:2n-6, alpha-linolenic acid (ALA) C18:3n-3

c AUC, the area under the curve values were calculated using GraphPad, based on all-time recorded data from each participant’s outcome measures.

### Statistical analysis

Multiple regression was performed using GraphPad Prism, Version 9.4. The least squares multiple regression method was used to assess whether genetic variation in each of the 25 SNPs significantly predicted participants’ physiological or subjective responses. The dependent variable (Y) represented the participant’s outcome measure or response variable, while the independent variable (X) or predictor variable represented the participant’s genetic variant, categorized as normal, high, or, in certain cases, low risk. During each test, the genetic variants were treated as categorical variables and encoded with a numerical code. Age and BMI were added as predictor variables. The F significance *P* value and the coefficient (β) *P* value were required to be ≤ 0.05 to be considered statistically significant. GraphPad assessed the normality and homogeneity of data using the D'Agostino–Pearson test. Of the data sets, the majority were normally distributed, and parametric statistical approaches were used. Transformation of nonnormal data did not alter the findings.

The baseline (premeal) plasma measures were used to assess vitamin D, iron, zinc, total cholesterol, LDL, HDL, TG, glucose, and insulin concentrations. The area under the curve (AUC) values were calculated for postprandial fatty acid changes (specifically, linoleic acid (LA) 18:2 n-6 and alpha-linolenic acid (ALA) 18:3 n-3) and appetite changes (using an online Visual Analog 100-point Scale for fat-taste perception, sugar preference, and hunger). All-time recorded data from each participant’s outcome measures were used to calculate these AUC values. GraphPad Prism was used to calculate the AUC using the trapezoidal method for time point differences, with adjustments made for baseline values [34].

In the absence of direct testing for dietary components impacting fat metabolism and body weight, we used BMI as an indirect measure to assess the physiological responses associated with gene variants. BMI serves as an indicator of body composition, which can be influenced by metabolic processes [47, 48, 49, 50, 51, 52, 53]. Within our study, there were 4 gene variants that met this criterion: the mitochondrial uncoupling protein 1 gene (*UCP1*) variant rs1800592, which is associated with fatty acid oxidation and body weight [47, 48]; the transcription factor 7-like2 gene (*TCF7L2*) variant rs7903146 involved in response to total fat intake and body weight [49, 50], the fat-mass and obesity-related alpha-ketoglutarate-dependent dioxygenase gene (*FTO*) variant rs9939609 related to how the body responds to fat intake and body weight [[50], [51], [52]], the peroxisome proliferator-activated receptor γ2 gene (*PPARγ2*) Pro12Alavariant rs1801282 associated with response to fatty acid intake and body weight [53]. Using BMI as a physiological response, we could indirectly assess the association between these gene variants and dietary components that affect fat metabolism and body weight.

The weight categories for BMI used in this study were: Underweight < 18.5 kg/m^2^, Healthy/Normal 18.5 to 24.9 kg/m,^2^ and Overweight 25 to 29.9 kg/m^2^ [54].

## Results

### Baseline data

Presented is a comprehensive description of the mean baseline demographics. These demographic data encompass age, ethnicity (Supplemental Table A.1), and other relevant factors characterizing the participants in our cohort. Additionally, we have provided detailed information regarding the mean fasting levels of key variables of interest, such as body weight and height, and other pertinent biomarkers. These data offer a clear snapshot of the study population at the beginning of the research, serving as a crucial reference point for our analysis (Table 3).

**TABLE 3:** Participant characteristics (30 healthy young males)

Baseline anthropometrics	Mean (*n* = 30)	Standard deviation
Age (y)	27.7	3.6
Body weight (kg)	76.6	10.0
Body height (cm)	176.6	5.8
BMI (kg/m^2^)	24.5	2.7
Systolic pressure (mmHg)	117.3	11.7
Diastolic pressure (mmHg)	75.7	9.0
Resting heart rate (beats/min)	67.4	10.0
Baseline blood biomarkers		
Essential fatty-acid C18:2n-6 (mmol/L)	119.1	65.7
Essential fatty-acid C18:3n-3 (mmol/L)	6.2	7.0
Baseline appetite scores		
Fat-taste perception	52.1	23.3
Sugar preference	48.0	28.1
Hunger	72.7	25.3

The study was completed by 30 healthy young males, and there were no adverse effects reported throughout the study.

### Outcome responses

The values in Table 4 represent all the collective outcome responses for the 30 participants at baseline (premeal) and the corresponding gene variants. Alternatively, when indicated by the AUC label, these values represent the changes from premeal to postmeal. The AUC values were calculated using data from each participant collected at specific time points.

**TABLE 4:** Genetic variants associated with physiological and subjective responses within our cohort (30 healthy young males)

Gene variant	Physiological & subjective responses^a^	Mean value of response	Standard deviation	Median value
CYP2R1 rs10741657 & GC rs2282679	Vit D ng/mL	19.70	8.76	17.26
SLC17A1 rs17342717	Iron μmol/L	17.22	6.09	16.73
HFE rs1800562 & rs1799945	Iron μmol/L	17.22	6.09	16.73
TMPRSS6 rs4820268	Iron μmol/L	17.22	6.09	16.73
TRF2 rs7385804 & TF rs3811647	Iron μmol/L	17.22	6.09	16.73
SLC30A3 rs11126936	Zinc mg/L	0.90	0.10	0.85
APOA5 rs662799	Total cholesterol mmol/L	4.60	0.73	4.41
ABCG8 rs6544713 & APOA2 rs5082	LDL mmol/L	2.91	0.69	2.84
ABCA1 rs1883025	HDL mmol/L	1.47	0.41	1.41
ANGPTL3 rs10889353	TG mmol/L	1.14	0.65	1.00
ADCY5 rs11708067	Glucose mmol/L	4.96	0.41	5.04
IRS1 rs2943641	Insulin μU/mL	7.72	4.13	6.89
FADS1 rs174546 & rs174547	Fatty Acid (LA) C18:2n-6 mmol/L AUC	222.09	125.18	184.10
FADS1 rs174546 & rs174547	Fatty Acid (ALA)C18:3n-3 mmol/L AUC	12.78	7.74	9.59
UCP1 rs1800592 & FTO rs9939609, TCF7L2 rs7903146 &PPARγ2 rs1801282	BMI kg/m^2^ BMI kg/m^2^	24.5 24.5	2.69 2.69	24.9 24.9
CD36 rs1761667	Fat-taste perception appetite questionnaire AUC	134.92	92.68	117.40
GLUT2 rs5400	Sugar preference appetite questionnaire AUC	116.49	104.07	76.88
NMB rs1051168	Hunger appetite questionnaire AUC	191.26	94.03	184.15

a Values are baseline (premeal), or if indicated by the AUC label, represents the area under the curve for the pre-post meal changes (*n* = 30). The AUC values were calculated using data collected at multiple time points for each participant. For fatty acids, the specific time points were t-pre, t60, t120, t180, and t240. For the appetite questionnaire, the specific time points were t-pre, t0, t30, t60, t120, t180, and t240, using an online survey with a 100-point scale.

### Genetic variants and associated responses

The qualitative genetic variants of each participant, along with their associated physiological and subjective responses, were documented (Supplemental Tables A.2–A.9). The appetite changes questionnaire covered questions related to fat-taste perception, sugar preference, and hunger. Data was collected at specific time points: t-pre, t0, t30, t60, t120, t180, and t240, using an online survey with a 100-point scale. The participants’ AUC calculations for fatty acids and postprandial changes in long-chain PUFA, linoleic acid (LA) C18:2n-6, and alpha-linolenic acid (ALA) C18:3n-3 were performed. Plasma samples for fatty acid analysis were collected at specific time points: t-pre, t60, t120, t180, and t240.

### Multiple regression analysis results

Notably, the variant rs2943641 of the insulin-receptor substrate 1 gene (*IRS1*) was significantly associated with elevated fasting insulin concentration (*R*^2^ = 0.639, F (3,26) = 15.34, β = 6.376, *P* = < 0.0001) in participants with CT and CC genotypes. The variant rs1800592 of the mitochondrial uncoupling protein 1 gene (*UCP1*) was significantly associated with increased BMI (*R*^2^ = 0.261, F (2,27) = 4.759, β = -2.764, *P* = 0.007) in participants with GG and GA genotypes. The variant rs1801282 of the peroxisome proliferator-activated receptor γ2 gene (*PPARγ2*) was significantly associated with increased BMI (*R*^2^ = 0.200, F (2,27) = 3.371, β = 3.291, *P* = 0.024) in participants with GG and GC genotypes. Table 5 presents the results of multiple regressions performed using GraphPad Prism (Version 9.4), along with data on the reference allele, alternative allele, and allele frequency retrieved from gnomAD [55] and NCBI [56]. Significant results are indicated by asterisks alongside the F significance *P* value and the coefficient (β) *P* value. To be considered statistically significant, both values must be ≤ 0.05.

**TABLE 5:** Multiple regression results performed using genetic variants and associated physiological or subjective responses (30 healthy young males)

Gene	SNP	Genetic variant^a^	Ref^b^ allele Alt^c^ allele or frequency	Physiological/subjective measures (β)	*R*^2^	F Significance *P* value^d^	Coefficient (β)*P* value
CYP2R1	rs10741657	AA normal GG or GA high	Ref A = 0.38 Alt G = 0.62	Premeal vitamin D ng/mL	0.18	*P =* 0.15	0.03∗
GC	rs2282679	TT or TG normal GG high	Ref T = 0.72 Alt G = 0.28		0.18	*P =* 0.15	0.03∗
SLC17A1	rs17342717	CC low CT normal TT high	Ref C = 0.92 Alt T = 0.84	Premeal iron μmol/L	0.15	*P =* 0.24	0.11
HFE (C282Y)	rs1800562	GG low AG normal AA high	Ref G = 0.95 Alt A = 0.05		0.15	*P =* 0.24	0.11
HFE (H63D)	rs1799945	CC Low GC normal GG high	Ref C = 0.86 Alt G = 0.14		0.15	*P =* 0.24	0.11
TMPRSS6	rs4820268	GG or GA normal AA high	Ref G = 0.46 Alt A = 0.54	Premeal iron μmol/L	0.11	*P =* 0.39	0.25
TFR2	rs7385804	CA normal CC or AA high	Ref C = 0.36 Alt A = 0.64		0.11	*P =* 0.39	0.25
TF	rs3811647	GA or GG normal AA high	Ref G = 0.67 Alt A = 0.33		0.11	*P =* 0.39	0.25
SLC30A3	rs11126936	AA or AC normal CC high	Alt A = 0.00 Alt C = 0.00	Premeal zinc mg/L	0.12	*P =* 0.34	0.84
FADS1	rs174547 & rs174546	TT normal CC or CT high risk	Ref T = 0.67 Alt C = 0.33	Fatty-acid change C18:2.n-6 C18:3.n-3 mmol/L	0.40	*P =* 0.00	0.55
					0.58	*P* < 0.0001	0.19
		
UCP1	rs1800592	AA normal GG or GA high	Allele freq. A = 0.27 G = 0.47	BMI kg/m^2^<	0.26	*P =* 0.02∗	0.01∗
FTO	rs9939609	TT or AT normal AA high	Ref T = 0.60 Alt A = 0.40	BMI kg/m^2^	0.04	*P =* 0.56	0.58
TCF7L2	rs7903146	CC or CT normal TT high	Ref C = 0.71 Alt T = 0.29	BMI kg/m^2^	0.03	*P =* 0.64	0.81
APOA2	rs5082	TT or TC normal CC high	Allele freq. not known	Premeal LDL mmol/L	0.21	*P =* 0.10	0.24
FTO	rs9939609	TT normal AA or AT high	Ref T = 0.60 Alt A = 0.40	BMI kg/m^2^	0.04	*P =* 0.56	0.58
PPARγ2	rs1801282	CC normal GG or GC high	Ref C = 0.90 Alt G = 0.10	BMI kg/m^2^	0.20	*P =* 0.05∗	0.02∗
APOA5	rs662799	TT normal CC or TC high	Allele freq. not known	Premeal total cholesterol mmol/L	0.13	*P =* 0.29	0.65
ABCG8	rs6544713	CC normal TT or TC high	Ref T = 0.30 Alt C = 0.70	Premeal LDL mmol/L	0.17	*P =* 0.19	0.90
ABCA1	rs1883025	CC normal TT or TC high	Ref C = 0.74 Alt T = 0.26	Premeal HDL mmol/L	0.23	*P =* 0.07	0.85
ANGPTL3	rs10889353	CC normal AA or CA high	Ref A = 0.68 Alt C = 0.32	Premeal TG mmol/L	0.26	*P =* 0.05	0.76
CD36	rs1761667	AA normal GG or GA high	Ref G = 0.48 Alt A = 0.52	Fat-taste perception changes	0.19	*P =* 0.14	0.49
GLUT2	rs5400	CC normal CT or TT high	Allele freq. n/a	Sugar preference changes	0.04	*P =* 0.78	0.61
NMB	rs1051168	GG or GT normal TT high	Ref G = 0.73 Alt T = 0.27	Hunger changes	0.10	*P =* 0.43	0.88
ADCY5	rs11708067	GG normal GA or AA high	Ref A = 0.79 Alt G = 0.21	Premeal glucose mmol/L	0.09	*P =* 0.47	0.88
IRS1	rs2943641	TT normal CT or CC high	Ref T = 0.34 Alt C = 0.66	Premeal Insulin μU/mL & BMI kg/m^2^	0.64	*P =* <0.0001∗	<0.0001∗ <0.0001∗

Each test was conducted using the least squares multiple regression. The dependent variable (Y) represented the participant’s outcome measure or response variable. The independent variable (X) or predictor variable represented the participant’s genetic variant, categorized as normal, high, or low risk. The genetic variants were treated as categorical variables and encoded with a numerical codes. Age and BMI were added as predictor variables.

Reference allele, alternative allele and frequency retrieved from gnomAD [55]; and NCBI [56], from population groups, European, African, African others, African American, Asian, East Asian, Other Asian, Latin American 1, Latin American 2, South Asian European.

a The qualitative genetic risk results were low, normal, or high. Low or normal risk indicates a low or normal response to a genetic variant and a high genetic risk indicates an increased response to a genetic variant.

b Reference (Ref) allele frequency is the base found in the reference genome and is not always the major allele.

c Alternative (Alt) allele frequency is the base found at the locus, other than the reference allele.

d The F significance *P* value and the coefficient (β) *P* value must be ≤ 0.05 to be considered statistically significant

## Discussion

This exploratory study investigated whether genetic variations were associated with interindividual differences in fasting levels of vitamin D, iron, zinc, LDL, HDL, total cholesterol, TG, glucose, and insulin concentrations, as well as postprandial fatty acid responses, appetite changes, and BMI in a cohort of 30 healthy young males. Physiological and subjective responses were assessed before and after a mixed meal intervention to explore potential associations with nutrient-related genes. Our finding revealed significant associations between 3 genetic variants and differences in physiological responses, particularly fasting insulin concentrations and BMI, within our cohort.

No significant associations were observed between the genetic variants and postprandial fatty acid responses, appetite changes, or the remaining fasting and BMI responses within our cohort.

The absence of significant results can be attributed to several factors, including the small sample size of male participants and the limited selection of SNPs obtained from the testing company Nutrigenomix. This highlights the challenges associated with investigating high-risk SNP variants characterized by low allele frequency within the general population. For instance, the minor G allele of the PPARγ2 gene variant rs1801282 was notably rare in our cohort, with only 3 participants carriers. The frequency of this G allele within population groups is 0.10 [55,56]. This scarcity makes it difficult to attain the necessary statistical power for detecting significant effects. To address this limitation and enhance statistical power, a substantial increase in participant enrollment would have been necessary. However, it is important to recognize that even with a larger enrollment, low carrier numbers with the required genotype may persist when the genetic variant is infrequent, thus posing a considerable hurdle in achieving the necessary statistical power for detecting effects.

In the absence of direct testing for some dietary components, BMI was utilized as an indirect measure for physiological responses associated with gene variants that affected fat metabolism and body weight [[47], [48], [49], [50], [51], [52], [53]]. These variants included *UCP1*, which is associated with fatty acid oxidation and body weight; *TCF7L2,* involved in the response to total fat intake and body weight; *FTO,* associated with the response to fat intake and body weight; and *PPARγ2,* associated with the response to fatty acid intake and body weight. Therefore, using BMI as a physiological marker proved valuable for exploring the association between dietary components and genetic variants affecting fat metabolism and body weight. The widespread use of BMI as an indicator of overall adiposity, along with its well-documented association with various health outcomes, made it a valuable physiological marker for our research [19, 37].

Previous research has reported significant associations between genetic SNPs and baseline or postprandial blood glucose concentrations, highlighting interindividual differences [57]. However, our study did not find any significant relationship between SNPs and TG or glucose concentrations. Similarly, our study found only a few genetic SNPs significantly associated with the tested physiological markers. These findings emphasize the complexity of health-related conditions, suggesting that relying solely on individual SNPs to fully understand physiological variables and their clinical relevance may oversimplify the process.

Numerous studies have provided evidence supporting the significance of metabolic efficiency and subtle genetic variations as independent risk factors for health and disease [22, 29, 30]. These factors, including genetics, may contribute to interindividual differences in both physiological and subjective responses during preprandial and postprandial states [22]. Therefore, examining the association between gene–nutrient interactions and potential disease susceptibility in individuals with specific genotypes remains an important area of research [21].

GWAS have identified specific genome regions where gene–nutrient interactions can have disease-causing effects, although the strength of these associations may vary [20]. For instance, the *ABCG8* gene variant rs6544713, T allele, has been associated with elevated LDL concentrations, which can contribute to conditions such as atherosclerosis and, eventually, coronary artery disease [58]. These effects are attributed to the elevation of cholesterol uptake and lowering of cholesterol secretion from the intestines [[59], [60], [61]]. Numerous studies have emphasized the need to carefully consider confounding factors such as diet and lifestyle choices, which can increase risk of cardiovascular diseases [62].

In another study, a meta-analysis combining 21 GWAS, using 46,186 nondiabetic European subjects, revealed that loci near the adenylate cyclase 5 (*ADCY5*) gene were associated with fasting glucose concentration [63]. Subsequent analysis, adjusting for BMI, demonstrated that the *ADCY5* gene was associated with high fasting glucose concentration of 0.027 mmol/L in A allele carriers (P = 0.0001). Consequently, A allele carriers had an increased risk of type 2 diabetes compared with G allele carriers. It should be noted, however, that studies differ regarding allele frequency, effect size, and the population they studied, and these associations only contribute to increased odds of disease occurrence [64, 65].

Confirming gene–nutrient associations with disease risk and providing dietary advice to individuals with risk variants remains a challenge [65]. The field of utilizing genetic information to guide dietary decisions is still in its infancy, and advancement has been slow due to limited evidence and a lack of study replication [66].

There was variability in the appetite and hunger questionnaires, which is common in self-reported questionnaire data. Although many publications reporting hunger and appetite utilize structural equation modeling to analyze variance data, we have presented our results in terms of standard deviations (SD) [67]. Nonetheless, these findings highlight the significance of gene–nutrient associations and their potential to advance our understanding of disease development and prevention.

In our study, we observed a significant association between the *IRS1* gene variant rs2943641 and elevated fasting insulin levels in participants with CT and CC genotypes. Several studies have linked insulin resistance and elevated insulin concentrations (outside of the normal range) to the *IRS1* gene, which plays a vital role in insulin signaling [68].

A population-based cohort study recruited 3344 Swedish participants born between 1923 and 1950 and 4905 Finnish participants [69]. The study searched for a link between nondiabetic participants and fasting insulin levels and identified a significant association (P = 2.4 × 10^-7^) between a location near the *IRS1* gene variant rs2943641 C allele and fasting insulin concentration. This study concluded that participants carrying the CT or CC genotype had greater fasting insulin concentration than those with the TT genotype. A comparable randomized control study conducted in Saudi Arabia, involving 376 type 2 diabetes patients and 380 healthy participants, reported similar results [70].

Within our cohort, it was observed that 15 participants with genotype CT or CC had higher than average fasting insulin concentration (>7.72 ± 4.13 μU/mL) than the other 15 with TT genotype, who had lower fasting insulin concentration, averaging < 7.72 ± 4.13 μU/mL. These findings suggest that carriers of genotype CT or CC are more likely to be at an increased risk of elevated fasting insulin concentration than genotype TT. This could potentially increase their susceptibility to noncommunicable diseases such as obesity, type 2 diabetes, and cardiovascular disease. Notable, insulin concentration and insulin resistance are affected by causal factors, including diet, lifestyle, and obesity [71].

In our study, a significant association was found between the *PPARγ2* gene variant rs1801282 GG and GC genotypes and participants’ BMI, which served as an indirect measure for physiological responses [53]. The *PPARγ2* gene polymorphism, specifically the Ala allele in Pro12Ala, has been reported to be associated with fat cell formation in adipose tissue, linked to insulin resistance, obesity, and BMI [53]. Researchers analyzed the *PPARγ2* polymorphism Pro12Ala (rs1801282) association with BMI and fat loss by studying 1,465 overweight and obese Spanish subjects who were placed on a controlled diet (89% completed the study) [53]. The study reported that subjects with genotype GG or GC had significantly lower obese levels with lower BMI (*P* = < 0.001) and had a more positive response to weight loss than genotype CC. The study concluded there was an association between the *PPARγ2* polymorphism Pro12Ala (rs1801282) and participants’ BMI.

Within our cohort, 3 participants were carriers of the *PPARγ2* gene variant (rs1801282) genotype GG or GC and had a BMI greater than 25 kg/m^2^. Due to the association between this gene variant and body weight, it is plausible that the 3 participants may achieve greater weight loss on a low-fat diet than those with genotype CC.

The *UCP1* gene is located on chromosome 4 of the human genome and is linked to fatty acid oxidation, energy expenditure, and body weight [72, 73]. In our study, BMI was used as an indirect measure to assess whether there was an association between the gene *UCP1* variant rs1800592 and body weight, and our findings were significant.

The *UCP1* gene encodes the *UCP1* protein, primarily found in brown adipose tissue, and facilitates proton transport across the mitochondrial inner membrane [47]. During *UCP1* activation, there is an increase in fatty acid oxidation to compensate for the decrease in ATP synthesis, and this allows energy expenditure to be maintained [48, 73]. Research suggests that the *UCP1* gene increases energy expenditure and decreases the mitochondrial membrane potential due to the *UCP1* gene polymorphism of rs1800592 (-3826 GA, -1766GA, and -112AC) in the intraperitoneal adipose tissue [48, 74, 75]. Several studies suggest that carriers of the *UCP1* gene polymorphism rs1800592 genotype GG or GA are at high risk of multifactorial diseases, including obesity, characterized by higher body weight and BMI, and type 2 diabetes. However, these findings have not been confirmed in all studies, with genotype AA often reported as having the lowest risk [48, 75].

Another study investigated the association between the *UCP1* gene variant rs1800592 and energy expenditure by examining 82 Japanese females aged 20 to 22 y, all from the same university campus in Japan [74]. The study compared energy requirements between the females with genotype GG or GA with the genotype AA females. The findings concluded that females carrying the G allele had a lower energy expenditure; therefore, their energy needs were lower than the AA genotype females.

According to our findings, there was a significant association between the *UCP1* gene variant rs1800592 and increased BMI. Specifically, participants with genotype GG or GA had a higher BMI compared with genotype AA. Within our cohort, 21 participants carried the GG or GA genotype, and 4 of them had a BMI greater than 25 kg/m^2^, classing them as overweight and at a higher risk of obesity [76]. This finding suggests that participants with genotype GG or GA may need to reduce their calorie intake while increasing their energy expenditure to lower their BMI, as their energy needs are lower than those of the AA genotype carriers [48, 74, 77].

Based on published articles, the best-matched genetic variant and physiological and subjective measures were chosen for this study. However, a significant limitation of our investigation is that some measures may be mismatched to the genetic variant as newly published scientific research is being produced. For example, while we associated hunger with the *NMB* gene, recent research has linked it to the *FTO* gene variant rs9939609, which is associated with postprandial sensation of hunger [78]. Additionally, since some dietary components could not be directly tested, we used BMI as an indirect measure of physiological responses associated with the genes *UCP1, TCF7L2, FTO,* and *PPARγ2.* These genes are involved in various metabolic processes, including fatty acid oxidation, response to total fat, protein, and fatty acid intake [[47], [48], [49], [50], [51], [52], [53]].

The traditional 6 h postprandial period was reduced to 4 h. Research suggests that most lipid changes occur within 4 h of a meal, particularly in males, capturing the peak lipid responses, with TG and cholesterol reaching their peaks within 2 to 4 h [38]. By ending the postprandial assessment after 4 h, the study has successfully balanced the capture of peak postprandial responses with practical considerations, thus reducing logistical challenges and participant burden.

The association between genetic variation, interindividual differences, and health benefits can be complex since other factors may also play a role. SNPs, for instance, are linked with other genetic risk variants, causing causal variants (linkage disequilibrium) and are affected by causal effects (lifestyle and environment) [79].

Eventually, gene–nutrient research may enable clinicians, dietitians, and nutritionists to tailor personalized dietary interventions based on an individual’s genetic information [80, 81]. However, practitioners should carefully consider the strength of the relationship between SNPs and associated outcomes before incorporating nutrigenomics into their practice [82].

In conclusion, this exploratory study found significant associations between 3 SNPs and differences in fasting insulin concentrations and BMI within our cohort, suggesting that gene–nutrient interactions might increase disease susceptibility for individuals with specific genotypes. However, our study did not yield significant associations between the genetic variants and postprandial fatty acid responses, appetite changes, or the remaining fasting and BMI responses. Despite our initial hypothesis, these results represent an exploratory investigation and suggest that further research with larger sample sizes or different genetic markers may be needed to fully understand the associations. By deepening our understanding of these associations, we can better appreciate their implications on health.

## Author contributions

The authors’ responsibilities were as follows—AB and JB: designed the research; TP, JB, and AB: conducted the research; TP, JB and HB conducted the acquisition of data, JB: conducted the interpretation of data and statistical analysis; JB, TP, and AB: wrote the manuscript; JB: had primary responsibility for the final content of the manuscript; and all authors provided content and feedback to the manuscript and read and approved the final manuscript.

## Funding

The wider research project was funded by the Meat Industry Association Innovation Limited, Beef and Lamb New Zealand Limited and the New Zealand Ministry of Business, Innovation and Employment. This research was internally funded by Faculty of M&HS. TP was funded by National Heart Foundation & Health Research Council.

## Data availability

Data described in the manuscript, code book, and analytic code will be made available upon request pending application and approval.

## Declaration of interests

The authors declare the following financial interests/personal relationships which may be considered as potential competing interests: Julie Brown reports financial support was provided by Meat Industry Association Innovation Limited. Andrea Braakhuis reports a relationship with Meat Industry Association Innovation Limited that includes: funding grants.

## Conflict of interest

The authors declare that they had no conflict of interest related to this study.
