# Supplementary material for: Specific Genotypes Associated with Differences in Fasting Insulin Levels and Body Mass Index in Healthy Young Males: Implications for Gene–Nutrient Interactions—an Exploratory Study
Source: Curr Dev Nutr. 2023 Oct 21;7(11):102018. doi: 10.1016/j.cdnut.2023.102018 (PMC10663744; doi:10.1016/j.cdnut.2023.102018)
Supplement: Multimedia component 1 [file mmc1.docx]

# Supplementary Data Specific genotypes’ associated with differences in fasting insulin levels and body mass index in healthy young men: implications for gene–nutrient interactions – an exploratory study

Brown et al.

**Appendix A:** **Tables A.1 – A.9 including** **Participant’s** **Ethnicities, Qualitative Genetic Variants** **and their Associated Quantitative Physiological or Subjective Outcome Measures, Postprandial Appetite and Fatty Acid Changes (30 healthy young men).**

**Table A.1** Ethnicities of Participants

| **Ethnicities** | **Total** |
| --- | --- |
| Chinese | 3 |
| European | 1 |
| Filipino | 1 |
| Greek Australian | 1 |
| Indian | 4 |
| Iranian | 1 |
| Korean | 3 |
| Latin American | 2 |
| New Zealand European | 8 |
| NZ European, Māori, Chinese | 1 |
| Others (American) | 1 |
| Singaporean Chinese | 1 |
| Sri Lankan | 1 |
| UK | 1 |
| Vietnam | 1 |
| Total | 30 |

**Table A.2** Participant’s **Qualitative Genetic-Risk** Variants and their **Corresponding** Plasma Measures at Baseline for Nutrients

| ID | Genetic variant | Output measure | Genetic variant | Output measure | Genetic variant | Output measure | Genetic variant | Output measure |
| --- | --- | --- | --- | --- | --- | --- | --- | --- |
|  | **Vit D CYP2R1 rs10741657, & GC rs2282679** | **Vit D ng/mL** | **Fe Overload SLC17A1 rs17342717, HFE C282Y rs1800562(H63D rs1799945)** | **Fe umol/L** | **Low Fe TMPRSS6 rs4820268 &TFR2 rs7385804 &TF rs3811647** | **Fe umol/L** | **Zinc SLC30A3 rs11126936** | **Zinc mg/L** |
| 1 | high | 12.35 | low | 14.25 | normal | 14.25 | high | 0.82 |
| 2 | high | 19.81 | low | 20.89 | normal | 20.89 | normal | 0.81 |
| 3 | high | 9.69 | low | 16.05 | normal | 16.05 | high | 0.91 |
| 4 | normal | 20.85 | low | 19.62 | high | 19.62 | high | 0.79 |
| 5 | high | 13.16 | low | 17.44 | high | 17.44 | normal | 0.80 |
| 6 | high | 12.72 | low | 16.62 | high | 16.62 | high | 0.74 |
| 7 | high | 20.53 | low | 9.36 | high | 9.36 | high | 0.85 |
| 8 | high | 24.11 | low | 16.84 | normal | 16.84 | normal | 0.74 |
| 9 | high | 16.99 | low | 15.83 | normal | 15.83 | normal | 0.80 |
| 10 | high | 8.40 | low | 14.37 | normal | 14.37 | normal | 0.93 |
| 11 | high | 21.54 | low | 12.61 | normal | 12.61 | high | 0.79 |
| 12 | high | 17.02 | low | 12.53 | high | 12.53 | high | 0.92 |
| 13 | high | 20.57 | low | 19.26 | high | 19.26 | normal | 1.10 |
| 14 | high | 25.73 | low | 13.72 | high | 13.72 | normal | 0.82 |
| 15 | high | 17.22 | low | 30.95 | normal | 30.95 | normal | 0.85 |
| 16 | high | 34.43 | low | 24.04 | normal | 24.04 | normal | 1.00 |
| 17 | high | 13.87 | low | 13.45 | high | 13.45 | normal | 0.69 |
| 18 | high | 13.27 | low | 25.37 | normal | 25.37 | high | 1.10 |
| 19 | high | 17.3 | low | 19.37 | normal | 19.37 | high | 0.92 |
| 20 | high | 28.65 | low | 17.26 | normal | 17.26 | high | 0.93 |
| 21 | high | 15.13 | normal | 18.11 | high | 18.11 | normal | 0.95 |
| 22 | normal | 19.72 | normal | 29.03 | normal | 29.03 | normal | 0.79 |
| 23 | high | 35.79 | low | 29.91 | high | 29.91 | normal | 1.00 |
| 24 | high | 11.81 | low | 9.06 | normal | 9.06 | high | 0.89 |
| 25 | high | 13.62 | low | 9.94 | high | 9.94 | high | 0.83 |
| 26 | normal | 48.86 | low | 21.18 | normal | 21.18 | normal | 0.86 |
| 27 | high | 10.54 | low | 7.52 | high | 7.52 | normal | 0.69 |
| 28 | high | 27.41 | low | 14.49 | high | 14.49 | normal | 0.94 |
| 29 | high | 17.12 | low | 17.64 | normal | 17.64 | normal | 0.97 |
| 30 | high | 22.77 | low | 9.87 | normal | 9.87 | high | 0.80 |

The qualitative genetic risk results were low, normal, or high. Low or normal risk indicates a low or normal response to a genetic variant and a high genetic risk indicates an increased response to a genetic variant.

**Table A.3** Participant’s **Qualitative Genetic-Risk** Variants and their **Corresponding** Plasma Measures at Baseline for Cholesterol, LDL, HDL and Triglycerides (30 healthy young men)

| **ID** | **Genetic variant** | **Output measure** | **Genetic variant** | **Output measure** | **Genetic variant** | **Output measure** | **Genetic variant** | **Output measure** |
| --- | --- | --- | --- | --- | --- | --- | --- | --- |
|  | **Tot. Chol APOA5 rs662799** | **Tot. chol mmol/L** | **LDL ABCG8 rs6544713** | **LDL mmol/L** | **HDL ABCA1 rs1883025** | **HDL mmol/L** | **TG ANGPTL3 rs10889353** | **TG mmol/L** |
| **1** | normal | 5.13 | normal | 3.47 | normal | 1.43 | high | 0.85 |
| **2** | high | 4.06 | normal | 2.81 | normal | 1.31 | high | 1.14 |
| **3** | normal | 3.21 | high | 1.58 | high | 1.52 | normal | 0.66 |
| **4** | normal | 4.74 | high | 3.46 | high | 1.43 | high | 0.80 |
| **5** | high | 4.55 | high | 3.30 | high | 1.23 | high | 0.61 |
| **6** | normal | 5.26 | normal | 3.71 | high | 1.29 | high | 1.02 |
| **7** | normal | 3.77 | normal | 2.49 | high | 1.21 | high | 0.72 |
| **8** | normal | 5.43 | normal | 3.62 | normal | 1.25 | high | 1.67 |
| **9** | normal | 4.40 | normal | 2.87 | high | 1.45 | high | 0.60 |
| **10** | normal | 4.03 | normal | 2.41 | high | 2.41 | high | 1.06 |
| **11** | normal | 5.24 | normal | 2.90 | normal | 2.47 | high | 0.57 |
| **12** | normal | 6.17 | normal | 4.49 | high | 1.48 | high | 1.05 |
| **13** | normal | 3.87 | normal | 2.12 | high | 1.69 | high | 0.81 |
| **14** | normal | 4.30 | high | 2.20 | normal | 2.06 | high | 0.59 |
| **15** | high | 4.39 | normal | 2.47 | normal | 1.39 | high | 1.90 |
| **16** | normal | 4.67 | high | 3.00 | normal | 1.68 | high | 0.57 |
| **17** | high | 5.05 | high | 3.48 | high | 1.08 | high | 1.79 |
| **18** | normal | 5.59 | high | 4.01 | normal | 1.06 | high | 1.68 |
| **19** | high | 3.96 | normal | 2.38 | high | 1.59 | high | 0.50 |
| **20** | high | 6.06 | high | 4.42 | normal | 1.21 | high | 1.25 |
| **21** | normal | 3.85 | high | 2.40 | normal | 1.19 | high | 1.16 |
| **22** | normal | 5.05 | normal | 3.27 | normal | 1.30 | high | 1.95 |
| **23** | high | 5.78 | normal | 2.88 | high | 2.14 | high | 1.43 |
| **24** | high | 4.42 | normal | 2.94 | high | 0.97 | high | 1.80 |
| **25** | high | 4.08 | normal | 2.62 | normal | 1.11 | high | 1.33 |
| **26** | high | 4.22 | normal | 2.49 | normal | 1.65 | high | 0.54 |
| **27** | normal | 4.25 | normal | 2.35 | normal | 1.73 | high | 0.98 |
| **28** | high | 4.25 | normal | 2.15 | normal | 1.85 | high | 0.73 |
| **29** | normal | 3.80 | normal | 2.41 | normal | 1.17 | high | 0.83 |
| **30** | normal | 4.55 | high | 2.59 | high | 0.66 | high | 3.59 |

The qualitative genetic risk results were low, normal, or high. Low or normal risk indicates a low or normal response to a genetic variant and a high genetic risk indicates an increased response to a genetic variant.

**Table A.4** Participant’s **Qualitative Genetic-Risk** Variants and their **Corresponding** Plasma Measures at Baseline for LDL, Glucose and Insulin (30 healthy young men)

| **ID** | **Genetic variant** | **Output measure** | **Genetic variant** | **Output measure** | **Genetic variant** | **Output measure** |
| --- | --- | --- | --- | --- | --- | --- |
|  | **Sat Fat APOA2 rs5082** | **LDL mmol/L** | **Glucose ADCY5 rs11708067** | **Glucose mmol/L** | **Insulin IRS1 rs2943641** | **Insulin uU/mL** |
| **1** | normal | 3.47 | high | 4.89 | high | 8.63 |
| **2** | normal | 2.81 | high | 3.64 | high | 3.94 |
| **3** | normal | 1.58 | high | 4.89 | high | 10.23 |
| **4** | normal | 3.46 | high | 5.22 | high | 8.69 |
| **5** | normal | 3.30 | high | 4.42 | high | 9.63 |
| **6** | normal | 3.71 | high | 5.59 | high | 17.25 |
| **7** | normal | 2.49 | high | 4.83 | high | 4.89 |
| **8** | normal | 3.62 | high | 5.11 | normal | 5.76 |
| **9** | normal | 2.87 | high | 4.67 | high | 8.10 |
| **10** | normal | 2.41 | high | 5.23 | high | 6.32 |
| **11** | normal | 2.90 | high | 4.56 | high | 4.76 |
| **12** | normal | 4.49 | high | 5.10 | high | 10.80 |
| **13** | normal | 2.12 | high | 4.83 | high | 13.15 |
| **14** | high | 2.20 | high | 4.81 | normal | 6.22 |
| **15** | normal | 2.47 | high | 5.46 | normal | 1.52 |
| **16** | normal | 3.00 | normal | 5.03 | high | 7.46 |
| **17** | normal | 3.48 | high | 5.05 | high | 16.43 |
| **18** | normal | 4.01 | high | 4.69 | normal | 3.28 |
| **19** | normal | 2.38 | high | 4.57 | high | 5.01 |
| **20** | normal | 4.42 | high | 5.13 | normal | 6.19 |
| **21** | normal | 2.40 | high | 4.86 | high | 7.59 |
| **22** | normal | 3.27 | normal | 5.04 | high | 11.50 |
| **23** | normal | 2.88 | high | 5.28 | high | 5.92 |
| **24** | normal | 2.94 | high | 5.32 | high | 12.83 |
| **25** | normal | 2.62 | high | 5.06 | high | 6.22 |
| **26** | high | 2.49 | high | 4.41 | normal | 1.29 |
| **27** | normal | 2.35 | high | 5.45 | high | 9.06 |
| **28** | normal | 2.15 | high | 5.17 | normal | 1.32 |
| **29** | normal | 2.41 | normal | 4.84 | high | 4.49 |
| **30** | normal | 2.59 | high | 5.65 | high | 13.12 |

The qualitative genetic risk results were low, normal, or high. Low or normal risk indicates a low or normal response to a genetic variant and a high genetic risk indicates an increased response to a genetic variant.

**Table A.5** Participant’s BMI used as the physiological marker to assess body composition and energy balance and their Corresponding **Qualitative Genetic-Risk** Variants (30 healthy young men)

**The categories of BMI were**: Underweight < 18.5 kg/m^2^, Healthy/Normal 18.5–24.9 kg/m^2^, Overweight 25–29.9 kg/m^2^, Obese > 30 kg/m^2^ (BMI retrieved from National Heart Foundation of New Zealand, 2022).

| **ID** | **Output measure** | **BMI** | **Genetic variant** | **Genetic variant** | **Genetic variant** | **Genetic variant** | **Genetic variant** |
| --- | --- | --- | --- | --- | --- | --- | --- |
|  | **BMI kg/m2** | **Classification** | **BMI**  **(energy balance)UCP1 rs1800592** | **BMI (protein)**  **FTO rs9939609** | **BMI**  **(total fat)**  **TCF7L2 rs7903146** | **BMI**  **(Sat.&unsat. fat) FTO**  **rs9939609** | **BMI (MUFA) PPARγ2 rs1801282** |
| 1 | 21.6 | Healthy | low | high | normal | high | normal |
| 2 | 22.7 | Healthy | low | normal | normal | normal | normal |
| 3 | 21.9 | Healthy | low | normal | normal | normal | normal |
| 4 | 22.3 | Healthy | low | high | normal | high | normal |
| 5 | 24.6 | Healthy | normal | normal | normal | high | normal |
| 6 | 26.8 | Overweight | low | normal | normal | normal | normal |
| 7 | 22.1 | Healthy | low | normal | normal | high | normal |
| 8 | 27.6 | Overweight | low | normal | normal | high | normal |
| 9 | 24.7 | Healthy | low | normal | normal | normal | normal |
| 10 | 22.5 | Healthy | low | normal | normal | normal | normal |
| 11 | 22.6 | Healthy | low | normal | normal | normal | normal |
| 12 | 24.7 | Healthy | low | normal | high | high | normal |
| 13 | 25.9 | Overweight | normal | normal | normal | normal | normal |
| 14 | 24.9 | Healthy | low | normal | normal | high | normal |
| 15 | 23.8 | Healthy | normal | high | normal | high | high |
| 16 | 25.2 | Overweight | normal | normal | normal | normal | normal |
| 17 | 29.1 | Overweight | normal | normal | normal | high | normal |
| 18 | 22.6 | Healthy | low | high | normal | high | normal |
| 19 | 23.3 | Healthy | low | normal | normal | high | normal |
| 20 | 31.3 | Obese | normal | normal | normal | high | high |
| 21 | 23.3 | Healthy | normal | normal | normal | normal | normal |
| 22 | 25.3 | Overweight | normal | normal | normal | normal | high |
| 23 | 22.3 | Healthy | low | normal | normal | normal | normal |
| 24 | 29.5 | Overweight | low | normal | normal | normal | high |
| 25 | 25.6 | Overweight | low | normal | normal | normal | normal |
| 26 | 24.1 | Healthy | low | normal | normal | high | normal |
| 27 | 20.3 | Healthy | low | normal | normal | normal | normal |
| 28 | 23.7 | Healthy | low | normal | normal | normal | normal |
| 29 | 21.6 | Healthy | low | normal | normal | high | normal |
| 30 | 29.2 | Overweight | normal | high | normal | high | normal |

The qualitative genetic risk results were low, normal, or high. Low or normal risk indicates a low or normal response to a genetic variant and a high genetic risk indicates an increased response to a genetic variant.

**Table A.6** Participant’s **Qualitative Genetic-Risk** Variant and their **Corresponding** Postprandial Changes in Long-Chain Polyunsaturated Fatty Acid (C18:3n-6, C18:3n-3) (30 healthy young men)

| **ID** | **Genetic variant** | **Output measure** | **Output measure** |
| --- | --- | --- | --- |
|  | **Lipids**  **FADS1 rs174547 & rs174546** | **Linoleic acid (LA)**  **C18:2n-6** | **α-linolenic acid (ALA)**  **C18:3n-3** |
| **1** | high | 123.00 | 5.53 |
| **2** | normal | 253.10 | 16.44 |
| **3** | normal | 153.90 | 7.83 |
| **4** | normal | 323.80 | 13.71 |
| **5** | high | 174.30 | 19.51 |
| **6** | high | 439.70 | 10.20 |
| **7** | normal | 162.70 | 7.47 |
| **8** | high | 278.70 | 18.12 |
| **9** | high | 135.40 | 7.95 |
| **10** | normal | 176.10 | 8.87 |
| **11** | high | 158.10 | 8.47 |
| **12** | normal | 192.10 | 22.24 |
| **13** | normal | 117.20 | 8.54 |
| **14** | high | 235.70 | 8.98 |
| **15** | normal | 26.18 | 2.80 |
| **16** | normal | 167.40 | 7.38 |
| **17** | high | 475.70 | 23.93 |
| **18** | normal | 349.10 | 15.32 |
| **19** | high | 27.10 | 2.57 |
| **20** | normal | 534.80 | 27.43 |
| **21** | normal | 398.80 | 25.90 |
| **22** | normal | 200.00 | 14.42 |
| **23** | high | 319.40 | 16.94 |
| **24** | normal | 234.40 | 19.59 |
| **25** | high | 172.70 | 10.21 |
| **26** | high | 113.10 | 2.94 |
| **27** | high | 75.27 | 4.65 |
| **28** | high | 221.00 | 7.00 |
| **29** | normal | 136.20 | 8.03 |
| **30** | high | 287.60 | 30.50 |

The qualitative genetic risk results were low, normal, or high. Low or normal risk indicates a low or normal response to a genetic variant and a high genetic risk indicates an increased response to a genetic variant.

**Table A.7** Participant’s **Qualitative Genetic-Risk** Variants and their **Corresponding** Appetite Changes using an Online Analogue 100-Point Scale (30 healthy young men)

| **ID** | **Genetic variant** | **Output measure** | **Genetic variant** | **Output measure** | **Genetic variant** | **Output measure** |
| --- | --- | --- | --- | --- | --- | --- |
|  | **Fat taste CD36 rs1761667** | **Fat taste change**  **yes 0 no 100** | **Sugar taste GLUT2 rs5400** | **Sweet taste change**  **yes 0 no 100** | **Hunger NMB rs1051168** | **Hungry change**  **no 0 yes very 100** |
| **1** | high | 199.80 | normal | 66.09 | normal | 368.00 |
| **2** | high | 112.50 | high | 87.00 | normal | 58.92 |
| **3** | high | 0.00 | normal | 0.00 | normal | 207.00 |
| **4** | normal | 80.25 | normal | 36.75 | normal | 347.50 |
| **5** | high | 152.80 | normal | 13.50 | normal | 185.30 |
| **6** | high | 273.00 | normal | 40.10 | normal | 96.67 |
| **7** | high | 76.08 | normal | 180.30 | normal | 55.26 |
| **8** | high | 122.30 | high | 150.50 | normal | 182.00 |
| **9** | high | 94.25 | normal | 314.00 | normal | 183.00 |
| **10** | high | 355.00 | normal | 248.50 | normal | 224.30 |
| **11** | high | 65.00 | normal | 321.80 | normal | 346.50 |
| **12** | high | 75.39 | high | 154.30 | normal | 251.80 |
| **13** | high | 234.30 | normal | 225.00 | normal | 265.00 |
| **14** | high | 229.00 | normal | 23.75 | normal | 114.20 |
| **15** | high | 262.50 | normal | 136.80 | high | 98.47 |
| **16** | high | 176.30 | normal | 61.76 | high | 229.30 |
| **17** | high | 44.25 | normal | 32.25 | normal | 126.70 |
| **18** | high | 39.00 | high | 260.00 | normal | 46.85 |
| **19** | high | 250.50 | normal | 28.34 | normal | 95.50 |
| **20** | normal | 124.70 | normal | 101.50 | normal | 143.50 |
| **21** | high | 35.00 | normal | 373.50 | normal | 265.50 |
| **22** | normal | 87.50 | normal | 55.00 | normal | 205.80 |
| **23** | high | 182.30 | normal | 167.80 | normal | 273.50 |
| **24** | high | 126.00 | normal | 123.50 | normal | 124.00 |
| **25** | high | 218.50 | normal | 20.88 | normal | 261.80 |
| **26** | high | 58.29 | high | 130.50 | high | 281.50 |
| **27** | high | 265.50 | high | 66.75 | normal | 337.50 |
| **28** | high | 37.50 | normal | 29.69 | normal | 158.80 |
| **29** | high | 44.25 | normal | 23.02 | normal | 97.75 |
| **30** | normal | 25.97 | normal | 21.77 | normal | 106.00 |

The qualitative genetic risk results were low, normal, or high. Low or normal risk indicates a low or normal response to a genetic variant and a high genetic risk indicates an increased response to a genetic variant.

**Table A.8** Participant’s appetite changes using an online 100-point scale and resulting area under the curve calculations (30 healthy young men)

| **Fat Taste** | **#1** | **#2** | **#3** | **#4** | **#5** | **#6** | **#7** | **#8** | **#9** | **#10** | **#11** | **#12** | **#13** | **#14** | **#15** |
| --- | --- | --- | --- | --- | --- | --- | --- | --- | --- | --- | --- | --- | --- | --- | --- |
| **Time** |  |  |  |  |  |  |  |  |  |  |  |  |  |  |  |
| **-1** | 51 | 70 | 100 | 79 | 49 | 13 | 27 | 50 | 50 | 21 | 100 | 51 | 40 | 21 | 40 |
| **0** | 95 | 100 | 100 | 100 | 87 | 67 | 37 | 100 | 21 | 100 | 100 | 77 | 74 | 82 | 90 |
| **0.5** | 99 | 94 | 100 | 100 | 96 | 84 | 11 | 95 | 36 | 99 | 100 | 76 | 87 | 77 | 100 |
| **1** | 100 | 100 | 100 | 97 | 90 | 93 | 24 | 87 | 50 | 100 | 100 | 77 | 91 | 72 | 100 |
| **2** | 88 | 81 | 100 | 100 | 84 | 80 | 46 | 75 | 20 | 100 | 100 | 63 | 97 | 71 | 100 |
| **3** | 97 | 100 | 100 | 98 | 87 | 67 | 50 | 50 | 25 | 100 | 66 | 64 | 100 | 74 | 100 |
| **4** | 97 | 99 | 100 | 80 | 39 | 45 | 71 | 31 | 29 | 100 | 38 | 45 | 100 | 49 | 100 |
| **AUC** | **199.80** | **112.50** | **0.00** | **80.25** | **152.80** | **273.00** | **76.08** | **122.30** | **94.25** | **355.00** | **65.00** | **75.39** | **234.30** | **229.00** | **262.50** |
|  |  |  |  |  |  |  |  |  |  |  |  |  |  |  |  |
| **Fat Taste** | **#16** | **#17** | **#18** | **#19** | **#20** | **#21** | **#22** | **#23** | **#24** | **#25** | **#26** | **#27** | **#28** | **#29** | **#30** |
| **Time** |  |  |  |  |  |  |  |  |  |  |  |  |  |  |  |
| **-1** | 31 | 82 | 88 | 35 | 54 | 92 | 40 | 46 | 72 | 50 | 94 | 41 | 50 | 39 | 42 |
| **0** | 83 | 89 | 91 | 100 | 100 | 100 | 100 | 100 | 100 | 100 | 97 | 100 | 100 | 48 | 33 |
| **0.5** | 87 | 95 | 91 | 100 | 100 | 100 | 50 | 100 | 100 | 97 | 100 | 100 | 50 | 50 | 41 |
| **1** | 95 | 94 | 95 | 100 | 100 | 100 | 50 | 97 | 100 | 94 | 91 | 100 | 50 | 49 | 47 |
| **2** | 80 | 94 | 100 | 96 | 76 | 100 | 50 | 85 | 100 | 100 | 95 | 100 | 50 | 48 | 46 |
| **3** | 44 | 92 | 100 | 87 | 52 | 100 | 50 | 77 | 100 | 100 | 67 | 100 | 50 | 49 | 48 |
| **4** | 29 | 85 | 100 | 50 | 33 | 98 | 60 | 59 | 100 | 99 | 43 | 100 | 50 | 50 | 22 |
| **AUC** | **176.30** | **44.25** | **39.00** | **250.50** | **124.70** | **35.00** | **87.50** | **182.30** | **126.00** | **218.50** | **58.29** | **265.50** | **37.50** | **44.25** | **25.97** |
|  |  |  |  |  |  |  |  |  |  |  |  |  |  |  |  |
| **Sugar Taste** | **#1** | **#2** | **#3** | **#4** | **#5** | **#6** | **#7** | **#8** | **#9** | **#10** | **#11** | **#12** | **#13** | **#14** | **#15** |
| **Time** |  |  |  |  |  |  |  |  |  |  |  |  |  |  |  |
| **-1** | 31 | 50 | 100 | 0 | 25 | 30 | 17 | 27 | 100 | 44 | 25 | 69 | 50 | 35 | 50 |
| **0** | 45 | 71 | 100 | 49 | 21 | 13 | 42 | 100 | 18 | 100 | 99 | 19 | 100 | 43 | 79 |
| **0.5** | 30 | 76 | 100 | 0 | 18 | 25 | 50 | 93 | 36 | 100 | 100 | 17 | 100 | 44 | 90 |
| **1** | 100 | 71 | 100 | 0 | 21 | 56 | 39 | 90 | 54 | 100 | 100 | 20 | 100 | 45 | 90 |
| **2** | 34 | 68 | 100 | 0 | 29 | 31 | 71 | 40 | 28 | 100 | 100 | 68 | 100 | 38 | 80 |
| **3** | 29 | 68 | 100 | 0 | 27 | 22 | 69 | 28 | 25 | 100 | 85 | 16 | 100 | 38 | 80 |
| **4** | 30 | 63 | 100 | 0 | 23 | 34 | 62 | 30 | 22 | 93 | 100 | 69 | 100 | 34 | 60 |
| **AUC** | **66.09** | **87.00** | **0.00** | **36.75** | **13.50** | **40.10** | **180.30** | **150.50** | **314.00** | **248.50** | **321.80** | **154.30** | **225.00** | **23.75** | **136.80** |
|  |  |  |  |  |  |  |  |  |  |  |  |  |  |  |  |
| **Sugar Taste** | **#16** | **#17** | **#18** | **#19** | **#20** | **#21** | **#22** | **#23** | **#24** | **#25** | **#26** | **#27** | **#28** | **#29** | **#30** |
| **Time** |  |  |  |  |  |  |  |  |  |  |  |  |  |  |  |
| **-1** | 50 | 22 | 82 | 36 | 91 | 17 | 50 | 91 | 72 | 25 | 100 | 49 | 20 | 49 | 33 |
| **0** | 78 | 29 | 31 | 42 | 100 | 100 | 100 | 6 | 100 | 9 | 95 | 64 | 40 | 23 | 21 |
| **0.5** | 65 | 34 | 29 | 50 | 100 | 100 | 50 | 11 | 95 | 20 | 90 | 75 | 15 | 50 | 30 |
| **1** | 70 | 36 | 25 | 39 | 100 | 100 | 50 | 67 | 100 | 24 | 39 | 67 | 20 | 49 | 32 |
| **2** | 58 | 30 | 13 | 50 | 77 | 100 | 50 | 56 | 100 | 23 | 72 | 58 | 15 | 50 | 33 |
| **3** | 60 | 23 | 31 | 32 | 53 | 100 | 60 | 82 | 100 | 22 | 73 | 63 | 10 | 51 | 42 |
| **4** | 39 | 25 | 17 | 37 | 17 | 100 | 65 | 87 | 100 | 30 | 58 | 61 | 23 | 50 | 19 |
| **AUC** | **61.76** | **32.25** | **260.00** | **28.34** | **101.50** | **373.50** | **55.00** | **167.80** | **123.50** | **20.88** | **130.50** | **66.75** | **29.69** | **23.02** | **21.77** |
|  |  |  |  |  |  |  |  |  |  |  |  |  |  |  |  |
| **Hungry** | **#1** | **#2** | **#3** | **#4** | **#5** | **#6** | **#7** | **#8** | **#9** | **#10** | **#11** | **#12** | **#13** | **#14** | **#15** |
| **Time** |  |  |  |  |  |  |  |  |  |  |  |  |  |  |  |
| **-1** | 100 | 80 | 80 | 99 | 49 | 90 | 40 | 80 | 88 | 50 | 100 | 95 | 100 | 60 | 80 |
| **0** | 18 | 16 | 80 | 0 | 6 | 90 | 24 | 2 | 48 | 1 | 10 | 95 | 40 | 19 | 20 |
| **0.5** | 6 | 81 | 0 | 0 | 6 | 0 | 19 | 7 | 53 | 0 | 0 | 0 | 56 | 20 | 100 |
| **1** | 0 | 78 | 0 | 0 | 5 | 95 | 25 | 12 | 58 | 0 | 14 | 34 | 52 | 21 | 10 |
| **2** | 9 | 74 | 0 | 0 | 8 | 81 | 50 | 74 | 50 | 0 | 20 | 34 | 25 | 40 | 90 |
| **3** | 12 | 81 | 73 | 67 | 7 | 69 | 36 | 50 | 41 | 0 | 30 | 29 | 39 | 45 | 85 |
| **4** | 89 | 71 | 40 | 62 | 18 | 43 | 80 | 80 | 32 | 0 | 71 | 32 | 48 | 62 | 80 |
| **AUC** | **368.00** | **58.92** | **207.00** | **347.50** | **185.30** | **96.67** | **55.26** | **182.00** | **183.00** | **224.30** | **346.50** | **251.80** | **265.00** | **114.20** | **98.47** |
|  |  |  |  |  |  |  |  |  |  |  |  |  |  |  |  |
| **Hungry** | **#16** | **#17** | **#18** | **#19** | **#20** | **#21** | **#22** | **#23** | **#24** | **#25** | **#26** | **#27** | **#28** | **#29** | **#30** |
| **Time** |  |  |  |  |  |  |  |  |  |  |  |  |  |  |  |
| **-1** | 80 | 24 | 50 | 35 | 86 | 62 | 71 | 90 | 30 | 100 | 100 | 100 | 80 | 64 | 19 |
| **0** | 14 | 61 | 92 | 0 | 80 | 0 | 20 | 0 | 0 | 0 | 11 | 0 | 40 | 11 | 54 |
| **0.5** | 10 | 98 | 0 | 0 | 74 | 0 | 10 | 0 | 0 | 1 | 72 | 10 | 0 | 49 | 44 |
| **1** | 13 | 91 | 51 | 0 | 74 | 0 | 15 | 20 | 0 | 5 | 13 | 20 | 35 | 50 | 20 |
| **2** | 23 | 11 | 50 | 26 | 52 | 0 | 20 | 31 | 10 | 86 | 21 | 30 | 70 | 47 | 29 |
| **3** | 50 | 15 | 51 | 44 | 29 | 7 | 40 | 50 | 1 | 70 | 58 | 40 | 50 | 48 | 49 |
| **4** | 65 | 35 | 51 | 59 | 20 | 13 | 45 | 71 | 0 | 56 | 71 | 45 | 50 | 50 | 72 |
| **AUC** | **229.30** | **126.70** | **46.85** | **95.50** | **143.50** | **265.50** | **205.80** | **273.50** | **124.00** | **261.80** | **281.50** | **337.50** | **158.80** | **97.75** | **106.00** |

AUC, the area under the curve values were calculated using GraphPad, based on all-time recorded data from each participant’s outcome measures. The specific time points were t-pre, t0, t30, t60, t120, t180, and t240.

**Table A.9** Participant’s postprandial changes in long-chain polyunsaturated fatty acid, linoleic acid (LA) C18:2n-6 and alpha-linolenic acid (ALA) C18:3n-3, and resulting area under the curve calculations (30 healthy young men)

| **LA C18:2n-6** | **#1** | **#2** | **#3** | **#4** | **#5** | **#6** | **#7** | **#8** | **#9** | **#10** | **#11** | **#12** | **#13** | **#14** | **#15** |
| --- | --- | --- | --- | --- | --- | --- | --- | --- | --- | --- | --- | --- | --- | --- | --- |
| **Time** |  |  |  |  |  |  |  |  |  |  |  |  |  |  |  |
| **0** | 83.42 | 69.67 | 35.49 | 70.46 | 144.06 | 124.58 | 64.87 | 207.21 | 72.33 | 101.26 | 144.52 | 132.39 | 90.64 | 188.37 | 115.78 |
| **1** | 165.81 | 99.63 | 64.71 | 120.44 | 155.64 | 114.24 | 96.42 | 264.04 | 153.87 | 115.90 | 234.12 | 153.51 | 116.24 | 201.40 | 110.03 |
| **2** | 104.08 | 176.61 | 61.34 | 144.55 | 270.04 | 191.35 | 117.98 | 293.99 | 116.03 | 157.75 | 189.94 | 186.63 | 134.02 | 106.79 | 110.74 |
| **3** | 98.03 | 146.01 | 116.74 | 171.09 | 112.16 | 413.45 | 101.24 | 344.49 | 80.16 | 160.26 | 141.87 | 224.08 | 143.05 | 100.06 | 115.78 |
| **4** | 94.02 | 149.33 | 70.72 | 268.58 | 83.55 | 289.84 | 148.13 | 202.58 | 77.05 | 193.24 | 195.40 | 182.46 | 61.83 | 60.25 | 85.00 |
| **AUC** | **123.00** | **253.10** | **153.90** | **323.80** | **174.30** | **439.70** | **162.70** | **278.70** | **135.40** | **176.10** | **158.10** | **192.10** | **117.20** | **235.70** | **26.18** |
|  |  |  |  |  |  |  |  |  |  |  |  |  |  |  |  |
| **LA C18:2n-6** | **#16** | **#17** | **#18** | **#19** | **#20** | **#21** | **#22** | **#23** | **#24** | **#25** | **#26** | **#27** | **#28** | **#29** | **#30** |
| **Time** |  |  |  |  |  |  |  |  |  |  |  |  |  |  |  |
| **0** | 53.26 | 123.53 | 99.58 | 92.30 | 35.84 | 151.12 | 176.41 | 152.12 | 162.16 | 138.18 | 143.15 | 169.69 | 6.12 | 72.25 | 351.02 |
| **1** | 80.92 | 324.41 | 175.76 | 90.99 | 211.15 | 210.07 | 245.06 | 227.63 | 263.74 | 137.51 | 181.85 | 173.80 | 15.69 | 114.78 | 416.60 |
| **2** | 86.25 | 230.79 | 218.61 | 75.39 | 158.02 | 283.80 | 279.66 | 224.97 | 241.54 | 179.43 | 213.68 | 210.07 | 108.53 | 111.25 | 418.71 |
| **3** | 158.92 | 256.36 | 237.04 | 91.96 | 215.92 | 291.47 | 197.52 | 255.63 | 200.40 | 234.09 | 122.04 | 182.38 | 86.14 | 116.52 | 417.99 |
| **4** | 55.50 | 192.97 | 132.34 | 75.22 | 150.39 | 284.75 | 134.32 | 287.15 | 192.59 | 209.30 | 159.58 | 205.86 | 64.12 | 93.01 | 525.74 |
| **AUC** | **167.40** | **475.70** | **349.10** | **27.10** | **534.80** | **398.80** | **200.00** | **319.40** | **234.40** | **172.70** | **113.10** | **75.27** | **221.00** | **136.20** | **287.60** |
|  |  |  |  |  |  |  |  |  |  |  |  |  |  |  |  |
| **ALA C18:3n-3** | **#1** | **#2** | **#3** | **#4** | **#5** | **#6** | **#7** | **#8** | **#9** | **#10** | **#11** | **#12** | **#13** | **#14** | **#15** |
| **Time** |  |  |  |  |  |  |  |  |  |  |  |  |  |  |  |
| **0** | 3.15 | 3.21 | 1.41 | 2.30 | 5.68 | 5.81 | 3.24 | 14.14 | 3.89 | 2.47 | 1.99 | 3.11 | 8.15 | 6.32 | 5.99 |
| **1** | 5.95 | 5.71 | 3.10 | 4.10 | 18.68 | 5.41 | 5.44 | 19.73 | 9.46 | 2.84 | 6.02 | 6.74 | 10.92 | 8.73 | 5.69 |
| **2** | 5.22 | 9.61 | 3.00 | 6.19 | 11.48 | 6.73 | 6.37 | 19.68 | 5.76 | 4.55 | 4.44 | 10.05 | 5.84 | 3.62 | 6.32 |
| **3** | 3.58 | 8.22 | 4.96 | 7.20 | 5.14 | 12.74 | 4.72 | 21.73 | 3.51 | 6.35 | 3.46 | 12.80 | 6.22 | 3.30 | 8.32 |
| **4** | 3.61 | 8.26 | 3.41 | 8.54 | 4.36 | 10.27 | 4.55 | 12.02 | 2.99 | 7.54 | 3.02 | 7.06 | 2.58 | 2.07 | 5.99 |
| **AUC** | **5.53** | **16.44** | **7.83** | **13.71** | **19.51** | **10.20** | **7.47** | **18.12** | **7.95** | **8.87** | **8.47** | **22.24** | **8.54** | **8.98** | **2.80** |
|  |  |  |  |  |  |  |  |  |  |  |  |  |  |  |  |
| **ALA C18:3n-3** | **#16** | **#17** | **#18** | **#19** | **#20** | **#21** | **#22** | **#23** | **#24** | **#25** | **#26** | **#27** | **#28** | **#29** | **#30** |
| **Time** |  |  |  |  |  |  |  |  |  |  |  |  |  |  |  |
| **0** | 1.98 | 7.36 | 3.54 | 3.83 | 1.76 | 7.23 | 9.10 | 5.25 | 14.64 | 7.34 | 2.83 | 7.56 | 0.46 | 2.49 | 38.51 |
| **1** | 3.92 | 24.89 | 7.68 | 5.50 | 10.99 | 11.76 | 14.18 | 8.48 | 26.53 | 7.54 | 4.06 | 10.01 | 1.15 | 5.01 | 52.74 |
| **2** | 3.71 | 9.58 | 9.02 | 4.04 | 9.22 | 16.16 | 15.37 | 11.44 | 19.93 | 9.57 | 4.50 | 9.16 | 4.23 | 5.01 | 44.53 |
| **3** | 5.74 | 11.51 | 8.69 | 3.85 | 10.03 | 16.12 | 12.45 | 10.49 | 13.75 | 13.55 | 2.72 | 7.39 | 2.35 | 5.18 | 44.22 |
| **4** | 1.87 | 7.42 | 4.64 | 2.46 | 6.69 | 14.32 | 7.93 | 9.81 | 10.07 | 10.48 | 3.05 | 6.39 | 1.75 | 3.08 | 47.58 |
| **AUC** | **7.38** | **23.93** | **15.32** | **2.57** | **27.43** | **25.90** | **14.42** | **16.94** | **19.59** | **10.21** | **2.94** | **4.65** | **7.00** | **8.03** | **30.50** |

AUC, the area under the curve values were calculated using GraphPad, based on all-time recorded data from each participant’s outcome measures. The specific time points were t-pre, t60, t120, t180, and t240.
